# The effect of genotype and traditional food processing methods on *in-vitro* protein digestibility and micronutrient profile of sorghum cooked products

**DOI:** 10.1371/journal.pone.0203005

**Published:** 2018-09-07

**Authors:** Dilooshi K. Weerasooriya, Scott R. Bean, Yohannes Nugusu, Brian P. Ioerger, Tesfaye T. Tesso

**Affiliations:** 1 Department of Agronomy, Kansas State University, Manhattan, Kansas, United States of America; 2 United States Department of Agriculture-Agricultural Research Service, Manhattan, Kansas, United States of America; 3 Ethiopian Institute of Agricultural Research, Addis Ababa, Ethiopia; University of Illinois, UNITED STATES

## Abstract

Sorghum (*Sorghum bicolor* (L.) Moench) is one of the principal staple for millions of people in sub-Saharan Africa serving as the main sources of protein. However, protein digestibility is low in sorghum and this may be affected by processing methods. In this study 15 sorghum cultivars and one variety each of maize (*Zea maize*) and tef (*Eragrostis tef*) all of Ethiopian origin were investigated for *in-vitro* protein digestibility (IVPD), activity and concentration of anti-nutritional factors and micro nutrient profile in raw flour and various cooked food samples. Kafirin composition content and composition was also determined from raw flour samples of the sorghum cultivars. IVPD was significantly different between genotypes with both maize and tef superior to sorghum both in cooked and uncooked state except for the high lysine genotype *Wetet Be-gunchie*. Cooking significantly reduced IVPD in all crops but had only minor effect in maize. Results revealed a highly significant interaction between genotype and food processing methods where, occasionally, genotypes with highest IVPD under one processing method ended up to be the lowest under another. Trypsin inhibitor levels had a significant and negative correlation with IVPD (r^2^ = 0.1), while changes in phytic acid concentration and intrinsic phytase levels during processing followed opposite trends to each other. Processing increased mineral levels by 20–44% for iron and 4–29% for zinc perhaps due to degradation of phytic acid. Results demonstrated that protein digestibility and the concentration of anti- nutritional factors varied widely depending on the food type. Identification of specific genotypes for a specific food product may help improve the nutritional quality of sorghum based foods.

## Introduction

An important food crop in the semi-arid tropics of Asia and Africa, sorghum [*Sorghum bicolor* (L.) Moench] is also a key feed grain in many other parts of the world. Though similar to other cereals in its grain composition [1], the nutritional quality of sorghum is impacted by several endogenous and exogenous factors that make its proteins less digestible than other cereals, especially when wet-cooked [2–7]. Past research efforts made to address this problem have focused mainly on understanding the role of protein body structure [8,9,10] and its chemistry [4,8] producing valuable information that served as foundation for numerous research initiatives.

Research to determine the effect of various food-processing methods and anti-nutritional compounds on sorghum protein digestibility has also been carried out. However, the impacts have been largely genotype dependent and studies based on limited number of genotypes may not provide the whole picture of the complexities surrounding sorghum protein digestibility. Anti-nutritional compounds identified to be of significant interest for protein digestibility include phytic acid (Myo-inositol-1,2,3,4,5,6-hexakisphosphate), trypsin protease inhibitors, and phenolic compounds such as tannins. These compounds are reported to interfere with protein digestibility in one way or another and limit its protein bioavailability [6,11]. Phytic acid (phytate) acts as the major storage form of phosphorous accounting for 1–5% by weight in legumes, cereals, oil seeds, and nuts [12,13]. While phytate-protein complexes make proteins less digestible, phytate also chelates iron and zinc hindering both macro and micronutrient bioavailability [14,15,16]. Hence, low phytate foods or food products with degraded phytate have been shown to improve iron absorption [16,17] and should also increase absorption of other micronutrients.

Protease inhibitors, are another important group of anti-nutritional factors undermining protein digestion in grains and food products. Due to their abundance in leguminous seeds, trypsin inhibitors have been extensively studied in legumes including soybean [18–21] and lima bean [22,23,24]. Protease inhibitors are also reported in several cereals including maize [25,26] wheat [27], barley [27,28, 29], rye [27], oats [30], rice [31,32] and sorghum [33,34]. Sorghum is known to possess three iso-forms of trypsin protease inhibitors labelled as inhibitor I through III based on their biochemical properties. Inhibitors I and II inhibit both trypsin and chymotrypsin; whereas inhibitor III is known to primarily inhibit chymotrypsin [34].

Food processing methods influence the bioavailability of nutrients in cereal grains either through altering the effects of anti-nutritional factors or due to chemical changes that may occur to the proteins and its complexes with other compounds [35–42]. The best-known example of this for sorghum is the decrease in protein digestibility when sorghum is wet-cooked [9]. Other forms of cooking such as dry heating (e.g. popping) also have a negative impact on protein digestibility but to a lesser degree [43,44]. Food processing techniques such as extrusion, fermentation, dry roasting, malting/germination also affects digestibility of sorghum proteins [45,46]. However, many of previous studies have used laboratory based cooking methods and investigated only limited number of genotypes and thus may not adequately the actual food products. Scaling up the research to include larger number of genotypes and food products typically produced and consumed by native consumers of the crop may provide not only a robust data but also array of information that may be of use for disentangling the complexity related to sorghum protein digestibility. Such information may also be of critical importance in guiding the choice of germplasm for breeding new cultivars suited for making specific food products or those for broader food applications. Therefore, the present study was aimed at evaluating the nutritional value of diverse sorghum cultivars of Ethiopian origin processed into various traditional food products consumed in that country. Specifically, the objective was to determine the dynamism of *in-vitro* protein digestibility (IVPD) and anti-nutritional compounds across various food products prepared from diverse sorghum genotypes and their interaction between genotype and food processing methods on the bioavailability of proteins and micronutrients.

## Materials and methods

### Genetic materials

Fifteen sorghum cultivars and one maize (*Zea mays*) variety (*Melkassa 1*) and one tef (*Eragrostis tef*) variety (*Boset*) of Ethiopian origin were used in this study. The list and protein profile of the sorghum cultivars included in the study is presented in Table 1. Sorghum genotypes included both key landraces and released varieties in major sorghum growing areas in Ethiopia. In selecting the cultivars, efforts were made to capture as much diversity as possible not only in terms of genetics but also in their region of adaptation, physical and chemical grain attributes, and agronomic characteristics (maturity, plant height).

**Table 1:** Total protein, total kafirin, percent gamma kafirin profile of sorghum genotypes as related to their raw flour IPD.

Genotype	Raw flour IVPD	Total protein content (%)	Total kafirin (mAu)	Percent γ- kafirin
Wetet Be-gunchie^¥^	75.1(±1.0)^a^	13.1(±0.07)^c^	17313(±468.4)^g^	4.1^g^
Degalit-Yellow	63.0(±2.4)^b^	9.9(±0.14)^g^	18608(±667.6)^f^	5.6^f^
Masugi-Yellow	60.9(±0.2)^bc^	9.8(±0.01)^g^	18412(±302.5)^f^	5.7^f^
Chiro	58.6(±1.8)^cd^	9.9(±0.01)^g^	17216(±1200.9)^g^	6.2^f^
Jigurte	56.8(±2.9)^de^	11.1(±0.07)^f^	18995(±21.9)^f^	5.8^f^
AL-70	50.2(±2.8)^f^	11.5(±0.07)^e^	21764(±822.8)^e^	6.0^e^
05MI5064	49.9(±1.0)^f^	12.8(±0.14)^c^	22570(±146.2)^e^	7.2^c^
Dagim	49.6(±1.2)^f^	12.8(±0.07)^c^	24026(±295.2)^d^	5.3^e^
76T1#23	44.6(±1.1)^g^	15.1(±0.21)^a^	26401(±761.3)^ab^	5.8^c^
Teshale	40.7(±1.8)^h^	14.7(±0.21)^b^	27389(±413.6)^a^	7.5^a^
Meko	40.5(±1.0)^h^	14.4(±0.14)^b^	25234(±83.4)^bc^	6.9^b^
IS9302	38.8(±1.3)^hi^	11.7(±0.07)^e^	22246(±430.5)^e^	5.8^e^
IESV92021-DL^†^	38.2(±0.8)^hi^	12.2(±0.14)^d^	22506(±242.9)^e^	6.2^d^
Melkam	36.2(±0.3)^i^	15.0(±0.14)^a^	26589(±83.5)^a^	6.7^b^
Seredo^†^	27.7(±0.3)^j^	15.2(±0.35)^a^	25114(±156.0)^cd^	5.3^de^
Mean	48.7	12.6	22292	6.0
LSD	2.8	0.3	1181.4	-

Least square means ± SE in each column followed by the same letter are not significantly different at P ≤ 0.05

†tannin containing cultivars

¥ High-lysine sorghum cultivar.

IVPD–*in-vitro* protein digestibility.

### Sample preparation

The food samples were prepared following the actual procedure used to by local community to prepare the different food products. Samples were prepared in the Food Science laboratory Melkassa Agricultural Research Center of the Ethiopian Institute of Agricultural Research (EIAR), at Nazareth, Ethiopia. Grains from each genotype were milled using a Cyclotec™ Cyclone sample mill (FOSS, Foss Allé 1, Hilleroed, Denmark) with a 0.5 mm sieve and the flour samples were stored in a zip-lock bags at 4°C until needed. The flour was used to prepare three different food products (fermented flat bread, unleavened bread and porridge) and a dry-cooked product was also prepared from an intact grain following procedures as described below.

Dry-cooking was performed by placing a cleaned and dried sorghum grain on a metallic pan. The pan was placed on a stove top and a medium heat was applied and this continued until the grain was cooked.

For the fermented flatbread, 200g of flour from each variety was mixed with 180 mL of water at room temperature (25°C) and kneaded for 5 min. Then a (5% on flour weight basis) pre-prepared starter yeast culture containing sufficient amount of water was poured (ten mL for each sample) on to small wells made on the surface the dough. The dough was left for 48 h to ferment at room temperature (25°C). Approximately 80g of fermented dough was mixed with 30 mL water at room temperature (25°C) followed by 200 mL boiling water and then mixed thoroughly for 1 min. The mixture was left at room temperature (25°C) until the temperature dropped to 45°C and the mixture was added back on the fermenting dough and mixed well. To this, 100 ml of water was added and the mixture was let to ferment for 3 hours at room temperature (25°C) until a foamy slurry was formed. The slurry was poured on a temperature-controlled plate pre-heated to 400°C in circular motions and covered to cook for 2 minutes.

Porridge was prepared by adding 125g of flour to 250 mL of clean boiling water followed by continuous heating at 96°C with stirring for 10 mins.

The unleavened flatbread was made in similar way as the leavened bread but not fermented. The flour was mixed with water at room temperature (25°C) and kneaded while adding water until a soft, non-sticky dough was formed. The dough was poured again in circular manner on to pre-heated clay griddle and cooked at 140°C for 10 min until a brown color was developed. All products were dried in an oven at 40°C for 24 hrs. The dry products were ground and packed in plastic zip-lock bags and along with the raw flour samples of all sorghum varieties as well as maize and tef were shipped to the USA for chemical analysis.

### Sample characterization

For all tests, ground samples of four different food products, fermented flatbread, unleavened flatbread, the dry-cooked product, porridge and the raw flour was analyzed in duplicate.

*In-vitro* protein digestibility (IVPD) was determined according to the pepsin digestibility method [47,48]. Protein content of undigested flour and digested residue was determined through nitrogen combustion using a LECO 628 Nitrogen Determinator (LECO Corporation, St. Joseph, MI, USA) according to AACCI method 46–30.01 [49] with a N to protein conversion factor of 6.25.

Tannin concentration of the raw flour and food samples was determined using HCl vannillin assay [50]. Measurement of phytic acid was done using a commercial colorimetric kit (Megazyme phytic acid assay kit, Megazyme, Bray Business Park, Bray, Co. Wicklow, Ireland). Intrinsic phytase levels in food samples were measured using an acidic phytase activity assay using direct incubation [51]. Total Fe and Zn of the samples were analyzed using nitric-perchloric acid digestion method [52]. Analysis for Fe and Zn was carried out using an Inductively Coupled Plasma (ICP) Spectrometer (Model 720-ES ICP, Optical Emission Spectrometer, Varian Australia Pty Ltd, Mulgrave, Vic Australia).

Trypsin inhibitor activity in food samples was analyzed as described previously [53]. Preliminary tests were conducted to evaluate the need to defat samples prior to analysis and samples were defatted using three one hour extractions with hexane (1g sorghum to 10 mL of hexane) followed by air drying. No significant differences in trypsin inhibitory activity between defatted and non-defatted samples was noted, so non-defatted material was used for routine analysis. Trypsin inhibitors were extracted using one gram of sorghum flour stirred with 15 ml of 100 mM sodium phosphate buffer (pH 7.6) for 4 hrs. The slurry was centrifuged at 1789 xg for 10 min under cold conditions (0–5°C). An aliquot of 250 uL of the supernatant was dialyzed against 100 mM sodium phosphate buffer (pH 7.6) using Tube-O-DIALYZER™ Micro 4kDa MWCO (Cat# 786–611, G-bioscience, St. Louis, MO, USA) following manufacturer’s instructions. Trypsin inhibitor activity was determined as mg of trypsin inhibited per gram of flour sample (mg g^-1^). Raw flour samples were analyzed for kafirin content and composition using RP-HPLC with a C18 column [54].

### Statistical analysis

Data were analyzed considering a 5x17 factorial arrangement in a randomized complete block design using mix model procedure, MIXED in SAS version 9.4 [55], where genotypes were treated as blocks and food processing methods as treatments. Replicates were treated as a random effect. Significant least square means were separated using Fisher’s protected least significant difference (LSD) test with the probability of type-I error (α) set at 0.05. The same α level was used to generate the Pearson correlation coefficients between measured parameters using GGally package (Schloerke, 2016) in R [56].

## Results

Protein and total kafirin profile of the 15 sorghum cultivars included in this study is presented in Table 1. The protein content among cultivars ranged from a low of 9.8% in one of the farmers’ variety *Masugi-Yellow*, to a high of 15.2% in the high tannin red variety, *Seredo*. The high lysine cultivar, *Wetet Be-gunchie*, has an intermediate protein content of 13.1%. Similarly, the cultivars also displayed wide variability for total kafirin with the lowest levels in variety *Chiro* and *Wotet Be-gunchie* and the highest in *Teshale* and *Melkam*. Likewise, the γ-kafirin content among cultivars ranged from 4.1% in *Wetet Be-gunchie* to 7.5% in variety *Teshale* which also had the highest total kafirin. *Wetet Be-gunchie* despite its intermediate protein content had the lowest amount of both total kafirin and γ-kafirin indicating that it carries significant portion of non-kafirin protein.

The analysis of variance for IVPD, micronutrient concentration and anti-nutritional compounds is presented in Table 2. The results showed significant (P ≤ 0.001) genotype, food product and genotype × food product interaction effects for IVPD, phytic acid and phytase activity. The effects of genotype and food product were also significant for trypsin inhibitor activity (TIA), and Fe and Zn concentration showing that both genotype and processing methods can alter bioavailability of nutrients from sorghum grains and food products. There was not significant genotype × food product interaction effect for TIA as well as Fe and Zn content.

**Table 2:** Analysis of variance of *in-vitro* protein digestibility (IVPD), anti-nutritional compounds and micronutrient concentration as affected by genotypes and food processing methods.

Source	df	IVPD	Phytic acid	Phytase	Trypsin inhibitory activity	Iron (Fe)	Zinc (Zn)
Replication	1	30.9	9.6 x 10^−5^	2.4 x 10^−4^	0.03	4.8	1.9
Food Product (FP)	4	26463***	0.4***	0.99***	22.9***	3952.6***	372.8***
Genotype (G)	14	2651.2***	1.9***	0.13***	9.8*	3583.5***	3387.9***
G × FP	56	3573.6***	0.3***	0.18***	24.2	6633.0	891.5
Error	74	3.9	3.6 x 10^−4^	1.1 x 10^−4^	0.35	85.6	11.4

*, **, *** represents statistical significance at P ≤ 0.05, 0.01 and 0.001, respectively.

IVPD–*in-vitro* protein digestibility.

Across genotypes, the mean IVPD from raw flour samples ranged from a low of 27.7% in the high tannin variety *Seredo* to 75.1% in the high lysine sorghum *Wetet Be-gunchie*, while maize and tef had 55.8 and 62.3%, respectively (Tables 1 and 3). Sorghum cultivars including *Masugi- Yellow*, *Degalit-Yellow*, *Chiro* and *Jigurte* had a raw sample IVPD scores comparable to that of maize indicating the potential for developing high digestible sorghum varieties with comparable feed value with to that of maize. However, the results show different picture when the samples were cooked. Sorghum IVPD decreased in food products with dry cooked food having relatively higher IVPD followed by fermented bread, and the unfermented wet-cooked products having the least IVPD (Table 3). However, the reduction across genotypes was not consistent in various food products though the high tannin *Seredo* was consistently shown to have the least IVPD in all food products. The high lysine *Wetet Be-gunchie* did not maintain higher IVPD in all food products. In dry cooked samples, varieties *Chiro*, *Teshale* and the high tannin IS9302 had higher IVPD of about 30% which was markedly lower than ~40% in maize. While *Meko* had higher IVPD of 24% in fermented bread which is again considerably less than 45.4 and 33.8% in maize and tef, respectively. The IVPD in the unfermented food products, porridge and unleavened bread, was higher in the high lysine *Wetet Be-gunchie* (26.6 and 19.1%, respectively) than tef and other sorghum genotypes but lower than that of maize.

**Table 3:** Genotype mean estimates for *in-vitro* protein digestibility (IVPD) assay resulted for raw flour and four different food products.

Genotype	Raw flour	Dry-cooked	Fermented flatbread	Porridge	Unleavened flatbread
Melkassa-2 (Maize)	55.8(±1.5)^eA^	39.9(±3.9)^aB^	45.4(±8.1)^aAB^	36.1(±1.5)^aB^	39.7(±1.5)^aB^
Boset (tef)	62.3(±1.0)^bA^	NA	33.8(±2.7)^bB^	22.7(±1.0)^bC^	16.9(±0.7)^cD^
Wetet Be-gunchie^¥^	75.1(±1.0)^aA^	29.3(±4.0)^c-fB^	17.8(±0.7)^c-gC^	26.6(±2.6)^bB^	19.1(±0.8)^bC^
Degalit-Yellow	63.0(±2.4)^bA^	24.1(±1.2)^f-hB^	21.6(±3.1)^c-fBC^	12.5(±0.1)^d-gD^	14.8(±0.8)^cdCD^
Masugi-Yellow	60.9(±0.2)^bcA^	30.9(±1.1)^b-dB^	15.5(±1.5)^f-hC^	12.8(±0.7)^d-gC^	13.7(±0.7)^d-fC^
Chiro	58.6(±1.8)^cdA^	34.8(±4.5)^abB^	22.4(±0.7)^c-eC^	15.4(±2.1)^c-eCD^	11.5(±0.7)^fgD^
Jigurte	56.8(±2.9)^deA^	29.8(±0.2)^b-eB^	22.2(±0.3)^c-fC^	10.7(±0.9)^f-hD^	13.8(±0.5)^d-fD^
AL-70	50.2(±2.8)^fA^	23.9(±1.0)^ghB^	14.1(±0.9)^ghC^	14.8(±0.2)^c-fC^	14.2(±0.6)^deC^
05MI5064	49.9(±1.0)^fA^	28.2(±3.5)^c-gB^	16.1(±2.2)^e-hC^	18.5(±0.4)^cC^	17.0(±0.2)^bcC^
Dagim	49.6(±1.2)^fA^	30.4(±3.7)^b-eB^	16.8(±6.7)^d-hBC^	11.8(±1.7)^e-hC^	15.0(±3.1)^cdBC^
76T1#23	44.6(±1.1)^gA^	25.3(±1.5)^e-hB^	19.2(±2.9)^c-gBC^	13.7(±3.5)^d-gC^	13.2(±0.9)^d-fC^
Teshale	40.7(±1.8)^hA^	31.5(±2.5)^bcB^	20.8(±1.0)^c-gC^	16.6(±0.7)^cdCD^	12.1(±0.3)^e-gD^
Meko	40.5(±1.0)^hA^	29.2(±0.4)^c-fB^	24.4(±1.7)^cB^	11.5(±3.0)^e-hC^	9.6(±1.2)^gC^
IS9302	38.8(±1.3)^hiA^	31.8(±3.0)^bcAB^	23.5(±4.3)^cdBC^	11.5(±3.1)^e-hC^	12.4(±1.4)^d-fC^
IESV92021-DL^†^	38.2(±0.8)^hiA^	25.8(±0.4)^d-hB^	21.4(±0.6)^c-fB^	7.2(±0.6)^hiD^	13.7(±2.4)^d-fC^
Melekam	36.2(±0.3)^iA^	21.3(±0.6)^hB^	20.5(±0.7)^c-gB^	9.1(±2.3)^g-iC^	12.6(±1.2)^d-fC^
Seredo^†^	27.7(±0.3)^jA^	20.9(±1.4)^hA^	10.6(±4.5)^hB^	5.9(±2.3)^iB^	6.3(±0.1)^hB^
Mean (sorghum only)	48.7	27.8	19.1	13.2	13.3
L.S.D.	2.8	5.3	6.1	4.3	2.3

Least square mean ± SE in the same column followed by the same lowercase letters are not significantly (P ≤ 0.05) different. Least square mean ± SE in the same row followed by the same uppercase letters are not significantly (P ≤ 0.05) different.

†tannin containing cultivars

¥ High-lysine sorghum cultivar.

NA–not available

In general, IVPD was reduced in processed foods with the degree of the reduction dependent on genotype. Table 4 shows the IVPD of cooked products as percentage of the raw flour. Reduction in IVPD of cooked products was common for all crop types with the effect being less in maize and tef compared to sorghum. Nevertheless, the extent of the reduction was markedly different between sorghum genotypes and food products. Accordingly, the IVPD in IS9302 and *Teshale* were least affected by dry cooking that they maintained 82 and 77.3%, respectively, of the IVPD of the raw flour. Whereas, the high lysine cultivar *Wetet Be-gunchie* and *Degalit-Yellow* maintained only 39 and 38.3%, respectively, of the digestibility of the raw flour. The relative IVPD of fermented bread among genotypes was lower but had similar trend with that of dry cooked product with IS9302 and *Meko* showing the least reduction by maintaining 60.7 and 60% IVPD of the raw flour, respectively, while again *Wetet Be-gunchie* and *Massugi Yellow* maintaining only 23.7 and 25.0%, respectively, of the IVPD of the raw sample. The difference among genotypes of the relative IVPD in unfermented products was relatively small compared to that of fermented bread and dry cooked product with cultivar *Teshale* still having the highest (40%) relative IVPD in porridge while *Degalit-Yellow* and *Jigurte* having the least, 19.9 and 18.9%, respectively. Similarly, IESV92021-DL and *Melekam* had relative IVPD of 35.9 and 34.9%, respectively while *Masugi-Yellow* and *Chiro* had 22.4 and 19.6% relative IVPD, respectively. In general, the data showed not only the significant effect of processing but also of the genotypes where their ranks changed according to food products. Another important observation is that digestibility of cooked products are not consistent with that of raw samples begging for the need to evaluate genotypes in cooked samples when the target is to enhance the crop as human food. Moreover, though generally lower, the difference among genotypes for IVPD of cooked samples is not as wide as in the raw flour except for few high tannin sorghums that continue to be lower. Given the little correlation between raw flour and cooked product IVPD observed in this and previous studies, and the strong association between protein content and IVPD, effort to enhance protein nutrition from sorghum based food products needs to pay as much emphasis to protein content as digestibility. In this study the low digestible sorghums such as *Teshale*, *Melkam* or *76T1#23* that had higher protein content but relatively lower raw flour IVPD may not be inferior in total protein bioavailability both in cooked and uncooked state. In other words, because IVPD is expressed as percentage it does not provide the amount of protein that become available up on digestion that bioavailability of proteins from high and low digestible sorghums may not be that different when protein content is accounted for. Actual protein availability predicted from the IVPD and protein content data for different cooked products show little difference between the high and low digestible sorghums.

**Table 4:** *In-vitro* protein digestibility of processed food products from the test genotypes expressed as percent of raw flour IVPD.

Genotype*	IVPD of cooked products as % of raw flour IVPD			
	Dry-cooked	Fermented flatbread	Porridge	Unleavened flatbread
Melkassa-2 (Maize)	71.6	81.4	64.7	71.1
Boset (tef)	N/A	60.5	60.5	30.3
Wetet Be-gunchie^¥^	39.0	23.7	35.4	25.5
Degalit-Yellow	38.3	34.4	19.9	23.5
Masugi-Yellow	50.8	25.4	21.0	22.4
Chiro	59.4	38.3	26.4	19.6
Jigurte	52.6	39.1	18.9	24.3
AL-70	47.5	28.2	29.4	28.2
05MI5064	56.6	32.2	37.1	34.1
Dagim	61.3	33.9	23.7	30.3
76T1#23	56.7	42.9	30.8	29.5
Teshale	77.3	51.0	40.7	29.7
Meko	72.0	60.0	28.5	23.6
IS9302	82.0	60.7	29.6	32.0
IESV92021-DL^†^	67.4	56.2	19.0	35.9
Melekam	58.8	56.6	25.1	34.9
Seredo^†^	75.5	38.0	21.4	22.5

†tannin containing cultivars

¥ High-lysine sorghum cultivar.

IVPD–*in-vitro* protein digestibility.

**Fig 1:**
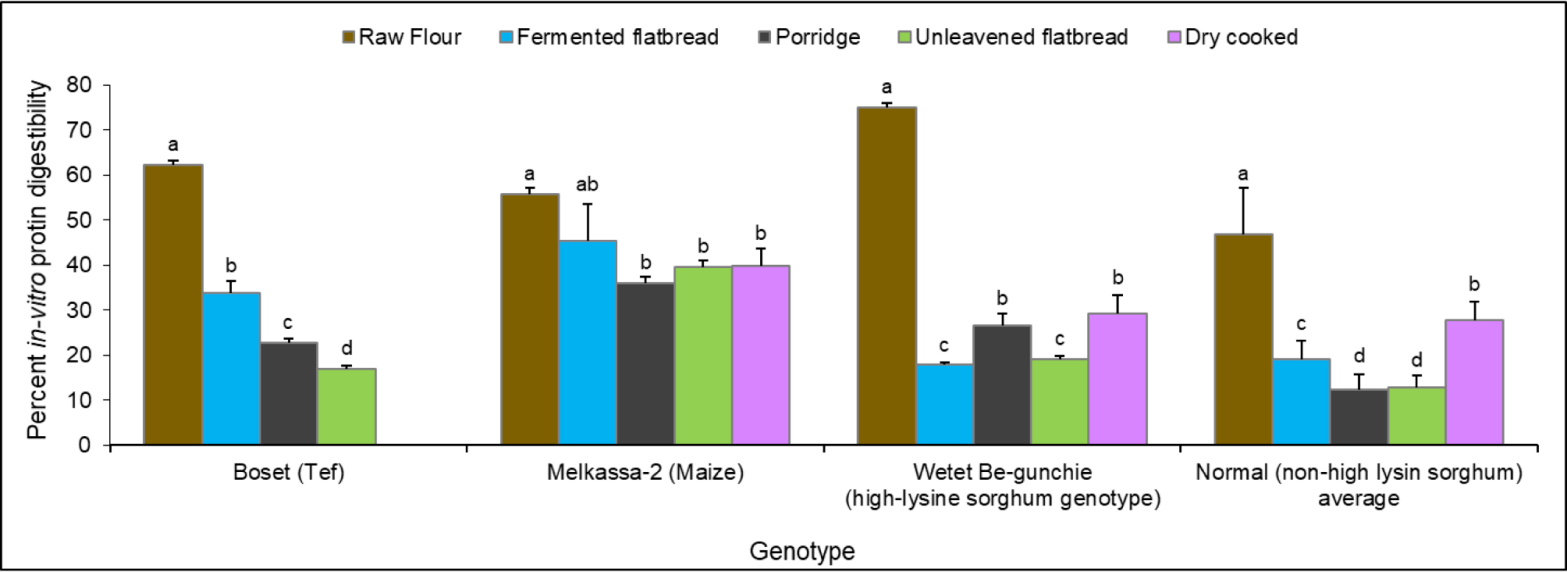
*In-vitro* protein digestibility (IVPD) across food products of maize, tef and high-lysine and average of 14 normal sorghum genotypes. Bars in each panel followed by the same letter are not significantly different.

A bar chart comparing the IVPD of two representative sorghum types, the normal and high lysine line, with that of maize and tef in different food products is presented in Fig 1. In all cases cooking had negative effect on IVPD in all crops, particularly tef and sorghum, but the effect clearly varies between food products. Maize was least affected by processing methods with IVPD in raw maize sample not significantly different from the fermented bread but lower in other food products (Fig 1). However, this was different in tef and sorghum where cooking had significantly reduced IVPD in all food products with the effect being more pronounced in sorghum. This shows that sorghum is not unique in the resistance of its protein to enzymatic degradation up on cooking. Additional understanding of the key factors responsible for the decrease in protein digestibility apart from disulfide crosslinking in the γ-kafirin fraction should be the subject of future investigation.

**Fig 2:**
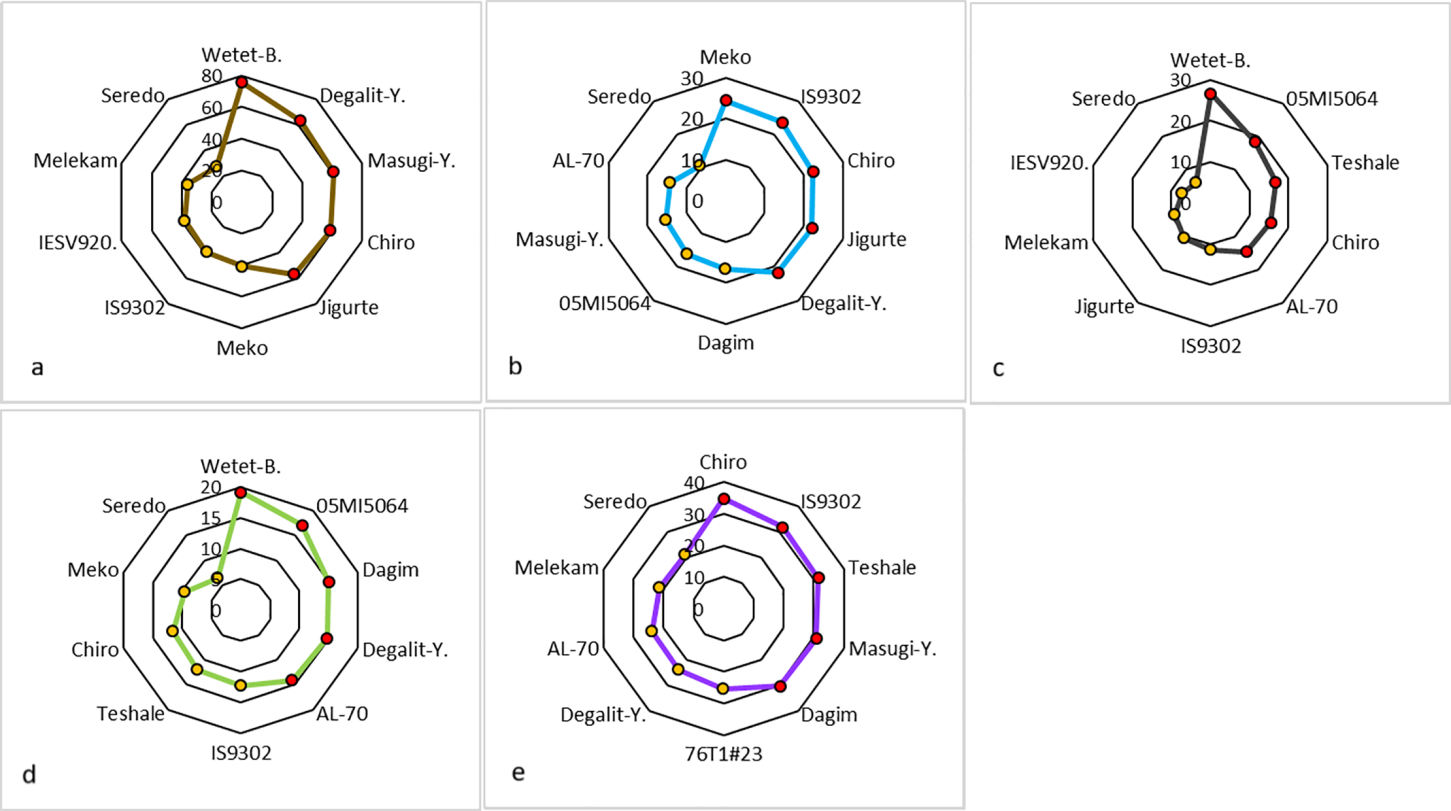
**Contour plots showing 10 sorghum genotypes with the highest (red dots) and lowest (yellow dots) *in-vitro* protein digestibility in (a) raw flour, (b) fermented flatbread, (c) porridge, (d) unleavened flatbread and, (e) dry-cooked sorghum**. Axis values in each plot depict protein digestibility range observed for each cooked product and raw flour.

Contour plots based on five genotypes with the highest and lowest IVPD were created in order to provide a graphical view of how IVPD varied between genotypes and food products (Fig 2). It was evident from the plot that the IVPD score of genotypes was specific to the food products. While the high-lysine sorghum genotype *Wetet Be-gunchie* had the highest IVPD for raw flour, porridge and unleavened bread, the genotype *Meko* was the highest for fermented flatbread and genotype *Chiro* for the dry cooked product. The high tannin genotype *Seredo*, however, had the lowest IVPD in all food products with porridge and unleavened bread having the least IVPD.

**Fig 3:**
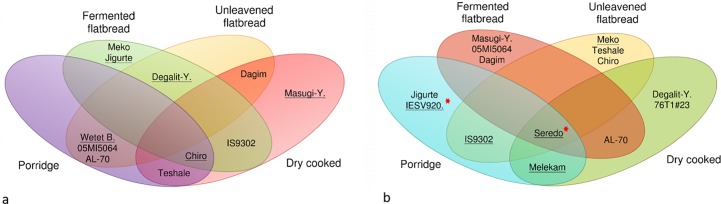
**Venn diagrams showing the distribution of sorghum genotypes with (a) highest and, (b) lowest IVPD score for cooked products**. The underlined cultivars are those highest or lowest IVPD score in raw flour. Tannin containing genotypes are marked with a star symbol.

The Venn diagram presented in Fig 3 shows the pattern of overlap for IVPD of the top five (Fig 3A) and bottom five (Fig 3B) genotypes tested in different food products. None of the cultivars consistently expressed high IVPD in all food products. However, many of the genotypes that had the highest raw flour IVPD were also among the highest in one or more of the food products. Cultivar *Chiro* was among the top five in three food products and was also among the highest in the raw flour. *Masugi-Yellow*, *Meko* and *Jigurte* were good only in one product. The other seven varieties were among the top in two products with five of them good for making fermented bread. On the other hand, all of the cultivars that were among the top five IVPD group for raw flour also had among the highest IVPD in one or more cooked products. Similarly, all cultivars that were among the lowest IVPD group in raw flour were also among the lowest IVPD group in one or more cooked products (Fig 3A and 3B and Table 3). Further, all of the cultivars that were among the highest IVPD group, except *Wetet Be-gunchie*, in one or more cooked products were also among the lowest IVPD group in one or more of the other cooked products. Whereas three of the lowest raw flour IVPD cultivars that were among the lowest IVPD group in one or more of cooked products were not among the highest IVPD group in any of the cooked products. Only *Seredo* was consistently grouped among the low IVPD category in all cooked products as well as the raw flour.

**Fig 4:**
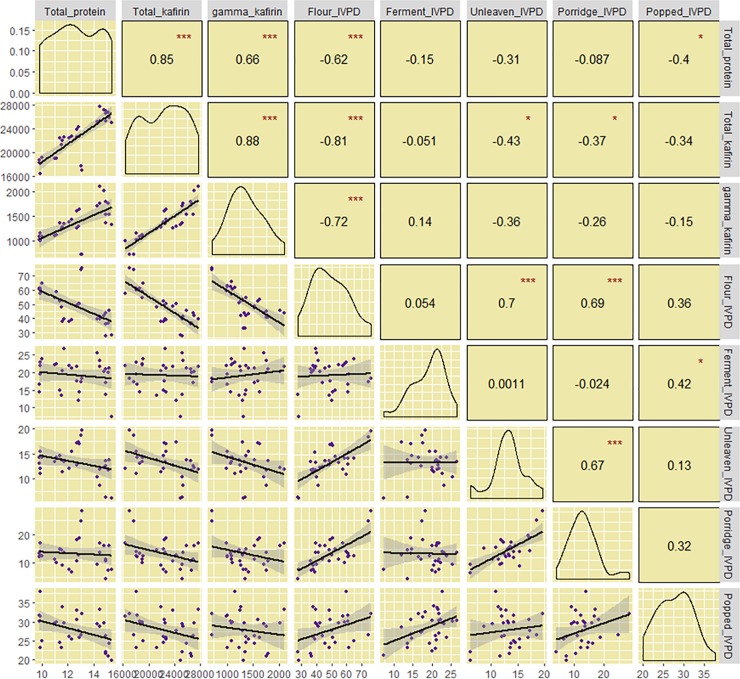
Correlation matrix for total protein content, total kafirin content, gamma-kafirin content and IVPD broken down based on food products and raw flour. Statistical significance at P ≤ 0.05, 0.01 and 0.001, are shown using *, **, ***, respectively.

As expected, there were positive correlations between total protein and total kafirin (r^2^ = 0.72) as well as total protein and γ-kafirin (r^2^ = 0.44) (Fig 4). Correlations of raw flour IVPD with total protein (r^2^ = 0.38), total kafirin (r^2^ = 0.66) and γ-kafirin (r^2^ = 0.52) were significant and negative as was IVPD of unleavened flatbread and porridge with total kafirin (r^2^ = 0.18 and r^2^ = 0.14, respectively). Raw flour IVPD showed positive correlation with porridge IVPD (r^2^ = 0.48) and unleavened flatbread (r^2^ = 0.49). Supporting this result was a positive correlation between IVPD of porridge and unleavened flatbread (r^2^ = 0.45). Likewise, IVPD in fermented bread and dry-cooked product also showed a positive correlation (r^2^ = 0.18).

Other grain characteristics may be related to IVPD, thus we evaluated the effects of phytic acid, Fe and Zn concentrations and the levels of phytase and trypsin inhibitor in the raw flour and cooked food samples on IVPD. Table 5 shows the estimate of these parameters in all product samples tested. As revealed by the data, mean phytic acid across cultivars was highest in the raw flour followed by the unfermented products (porridge and unleavened bread), and lowest in the fermented bread while the intrinsic phytase activity was highest in fermented bread and lowest in porridge and unleavened bread. Trypsin inhibitor activity was significantly lower in raw flour and increased during food processing. Fe and Zn contents showed considerable variability among food products while phytate/Fe and phytate/Zn molar ratios showed a consistent pattern with raw flour > porridge > unleavened flatbread > dry-cooked > fermented flatbread (Table 5). Fermentation appeared to have positive effect on enhancing protein nutrition in that it considerably reduced phytic acid and TIA, and significantly improved phytase activity (Table 5).

**Table 5:** Across genotype mean estimates for all measured parameters and phytate/mineral molar ratios in raw flour and four different food products.

Food Product type	Phytic acid (g/100g)	Phytase activity (Units/g)	TIA (mg/g)	Fe (mg kg^-1^)	Zn (mg kg^-1^)	Phytate/Fe molar ratio	Phytate/Zn molar ratio
Flour	0.77(±0.1)^a^	0.28(±0.1)^c^	6.27(±0.4)^c^	37.1(±7.2)^c^	22.0(±10.2)^b^	17.9^a^	35.4^a^
Dry-cooked	0.69(±0.1)^c^	0.38(±0.1)^b^	7.15(±0.6)^a^	48.5(±9.4)^a^	26.3(±12.5)^a^	12.3^c^	27.1^c^
Fermented bread	0.62(±0.1)^d^	0.44(±0.1)^a^	6.81(±0.9)^b^	52.7(±7.2)^a^	24.6(±11.2)^ab^	10.0^d^	25.1^d^
Porridge	0.75(±0.1)^ab^	0.23(±0.1)^d^	7.01(±0.9)^ab^	46.9(±13.4)^ab^	22.7(±12.2)^b^	14.2^b^	33.9^a^
Unleavened bread	0.72(±0.1)^bc^	0.25(±0.1)^cd^	7.16(±0.6)^ab^	45.5(±14.0)^b^	25.0(±11.9)^ab^	14.0^b^	29.4^b^
Mean	0.71	0.32	6.88	46.1	24.1	13.7	30.2
L.S.D.	0.06	0.021	0.33	5.1	3.36	1.4	2.5

Least square means ± SE in the same column followed by the same letter are not significantly different at P ≤ 0.05.

TIA–Trypsin inhibitory activity.

**Fig 5:**
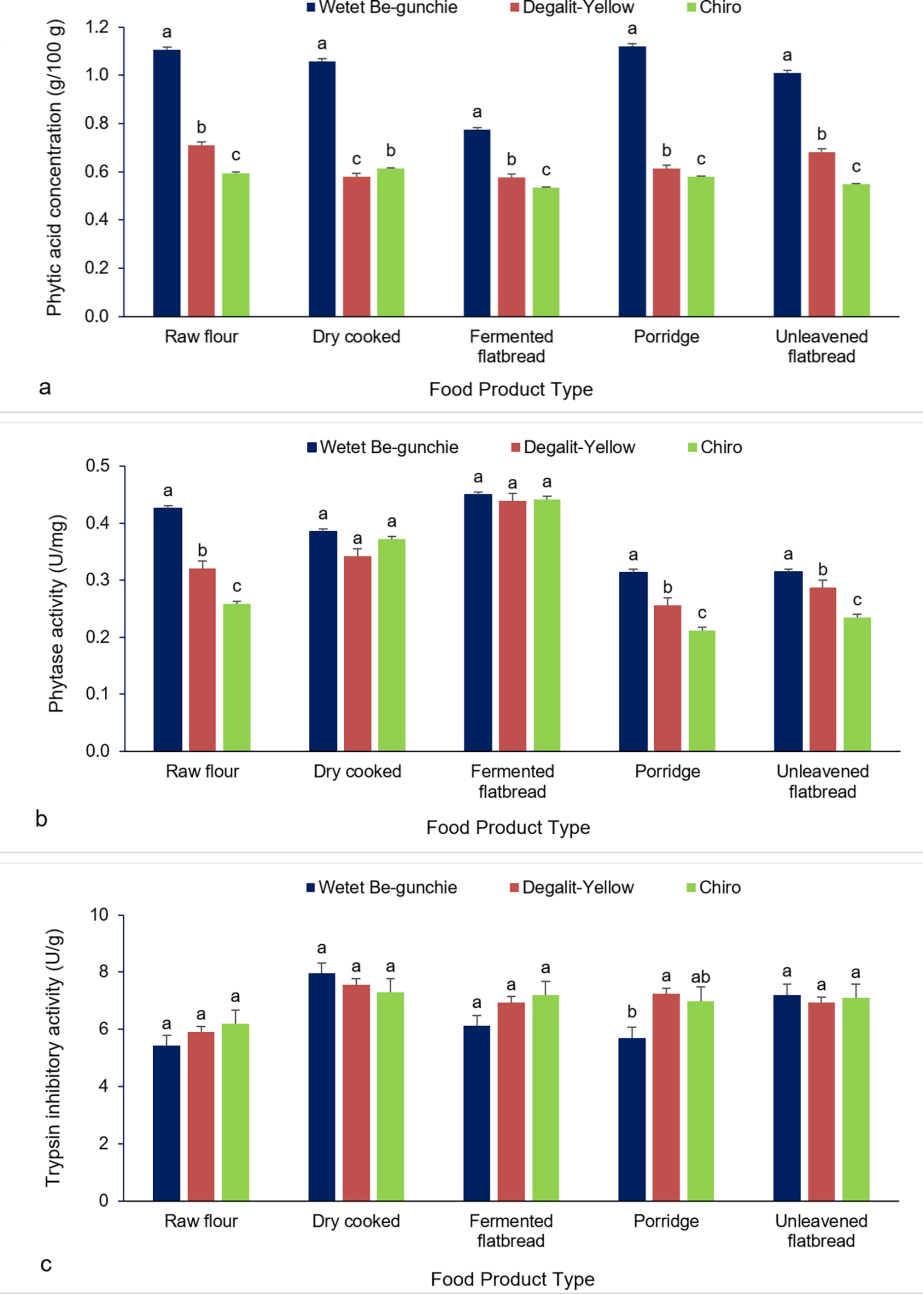
**Levels of anti-nutritional factors and intrinsic phytase levels in three sorghum cultivars shown to have among the highest IVPD in raw flour as well as in three or more cooked products: (a) phytic acid concentration, (b) phytase concentration and, (c) trypsin inhibitor levels**.

Fig 5 displays the profile of anti-nutritional factors and intrinsic phytase levels in sorghum cultivars shown to have high IVPD in two or more cooked products as well as raw flour. Phytic acid concentration was consistently and significantly higher in *Wetet Be-gunchie* in both raw flour and all of the cooked products, with the levels slightly lower in the fermented flat bread (Fig 5A). The two other cultivars, *Degalit-Yellow* and *Chiro* not only had markedly lower phytic acid concentration than *Wetet Be-gunchie* but also were different from each other with *Degalit-Yellow* having higher concentration than *Chiro*. *Wetet Be-gunchie* also showed higher levels of phytase activity in all products except in the fermented flat bread and dry-cooked product where the difference was not significant (Fig 5B). Again *Degalit-Yellow* had significantly higher phytase levels than *Chiro* in raw flour as well as in unfermented food products (porridge and unleavened bread). Trypsin inhibitor on the other hand was not significant between the three genotypes except in the cooked product porridge where *Wetet Be-gunchie* was shown to be significantly lower than the other genotypes (Fig 5C). In general, phytic acid concentration showed similar variability between genotypes in all food products with *Wetet Be-gunchie* containing the highest concentration (Fig 5A). Phytase activity was still higher in *Wetet Be-gunchie* but significant only in raw flour and unfermented products (Fig 5B). Trypsin inhibitor, however, was not significantly different except in porridge where *Wetet Be-gunchie* had the lowest activity (Fig 5C).

**Fig 6:**
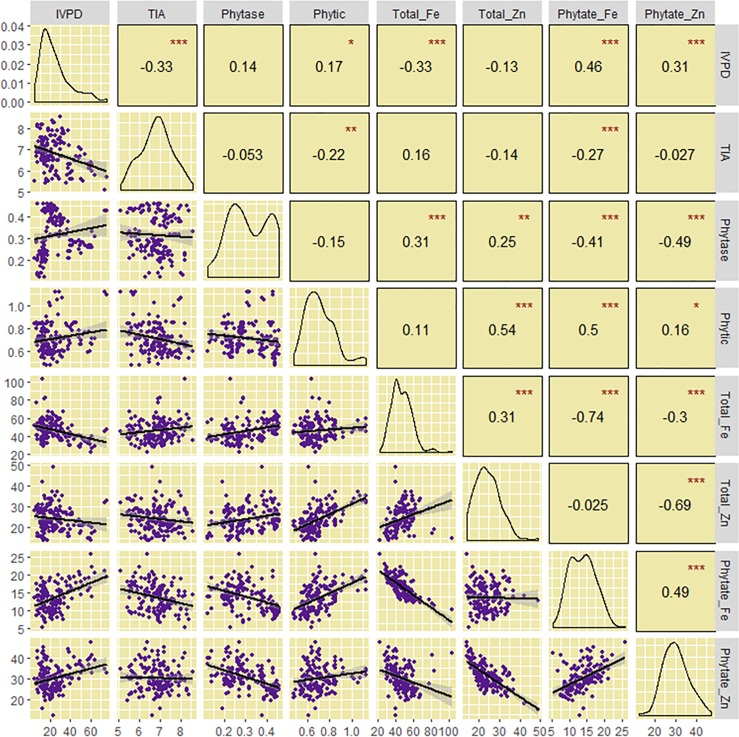
Correlation between IVPD, anti-nutritional factors (phytic acid and trypsin inhibitor), phytase concentration, and micronutrient content in raw flour samples of fifteen sorghum cultivars. *, **, ***, = statistically significant at P ≤ 0.05, 0.01 and 0.001, levels of probability, respectively.

Fig 6 shows the correlations between anti-nutritional factors, phytase activity, mineral concentrations and phytate/mineral molar ratios. While the raw flour IVPD showed significant and positive correlation with phytic acid (r^2^ = 0.03), trypsin inhibitory activity showed a significant and negative correlation with IVPD (r^2^ = 0.11). Correlations of phytase with Fe (r^2^ = 0.10) and Zn content (r^2^ = 0.06), and Fe with Zn (r^2^ = 0.10) were positive. Moreover, phytic acid showed a negative correlation with trypsin inhibitory activity (r^2^ = 0.05). Phytate/Fe and phytate/Zn molar ratios, on the other hand, showed positive correlation with IVPD (r^2^ = 0.21 and r^2^ = 0.10, respectively) and phytic acid (r^2^ = 0.25 and r^2^ = 0.02, respectively) and negative correlation with phytase (r^2^ = 0.16 and r^2^ = 0.24, respectively).

## Discussion

The significance of the discoveries of the effect of sorghum protein body structure [6,8,57] and disulfide cross-linking [4,7,58] on protein digestibility have received significant research attention in the past several years. Prior to that, the discovery of the Ethiopian high lysine landraces and the later discovery of the high lysine mutants had both attracted similar interest and the research work that followed has generated significant information on the nutritional quality of sorghum [59,60,61]. An equally important topic that has not attracted as much research attention is the impact of food processing on bioavailability of sorghum proteins. While not a new topic in sorghum utilization studies, research on local food processing methods, especially in relation to its potential outcome for enhancing the nutrition of smallholder households dependent on the crop. The goal of the current study was not to elucidate the mechanisms of how food processing impacts sorghum biomolecules, but rather to demonstrate the impacts of traditional food processing methods by native consumers of the crop on availability of protein and micronutrients. An additional outcome of this research was to identify areas where the science of food chemistry can be applied to add value to these processes and improve nutritional value of sorghum-based diets.

### IVPD is genotype and food product specific

As reported in several previous findings [47,62,63], the IVPD of sorghum significantly reduces when cooked to various food products and this was true for every genotype (Table 2). This was also true for tef as well confirming previous findings [9,47,62] while the reduction in IVPD of maize cooked products was substantially lower (Fig 1). The mean IVPD across genotypes was reduced in all food products compared to the raw flour. But the extent of the reduction was different for different food products with the mean IVPD ranking of raw > dry-cooked > fermented bread > porridge and unleavened bread and agrees with recently reported results [64]. Earlier studies have reported popping or roasting to have no negative effect on sorghum IVPD [43] but in this study it did affect IVPD in all sorghum varieties as well as in maize genotype. Several other investigations have shown fermentation to increase the IVPD of sorghum [39,65–67] compared to unfermented wet-cooking such as in unleavened bread and porridge which severely reduces digestibility [9,62,63] as is also the case in the current study.

However, IVPD showed unpredictable fluctuations when the flour was processed into various food types (Table 1 and Fig 2) even for genotypes that belonged to either of a known high or low uncooked IVPD groups (Figs 2 and 3). In other words, the pattern of the reduction in IVPD across genotypes for different food products was not consistent and rarely depended on that of raw flour IVPD. The only exception was the high tannin genotypes that had consistently lower IVPD in all food products as well as the raw sample. At the same time there was significant differences in IVPD of food products made from different genotypes but the differences did not show a consistent pattern. This could be explained by the significant genotype × food type interaction (P <0.001) observed for IVPD (Table 1).

The best demonstration of the observed genotype × food type interaction effect on IVPD was that of the high-lysine sorghum genotype *Wetet Be-gunchie* which had the highest IVPD for raw flour, porridge and unleavened bread, all of which are wet cooked products. However, this genotype did not fall among the highest IVPD for fermented flatbread and dry-cooked product (Table 2 and Fig 2). Other genotypes showed similar variation but not necessarily the same pattern. This result depicts a very important characteristics of sorghum in general where cooking not only decreased IVPD significantly but also unpredictably. The difference in IVPD of raw flour and cooked products has complicated the cultivar improvement process rendering the standard raw flour digestibility results irrelevant for food value. Added to this complication was the unpredictable difference in IVPD of various food products made from different varieties. This result explains why traditional farmers sort varieties not just by their grain color or agronomic value but also by the quality of food products they make. In Ethiopia, an ancient country where sorghum was believed to have originated, local farmers categorize the crop based on its utilization traits; they have sorghum types specifically used for making popped snacks, others for making bread and still others for local brew giving them distinct names that describes their use. This and previous research results suggest that sorghum improvement efforts for food use needs to take into account the intended use of the crop and conduct tests and selections for specific food products. Since food types and products vary from place to place depending on the culture of the people, the process of cultivar development may become complicated due to varying needs of communities.

The other important result was the association between IVPD and the content of grain protein and protein fractions. There was a significant negative correlation between IVPD and total protein, total kafirin as well as gamma kafirin content in raw flour samples (Fig 4). The negative association between γ-kafirin and IVPD agrees with the previous reports that γ-kafirin is resistant to pepsin owing to its high cysteine content that leads to disulphide cross-links [63,68,69]. The fact that *Wetet Be-gunchie*, the high lysine genotype with high protein digestibility also had low γ-kafirin agrees with the previous reports.

However, when the flour was cooked to different food products, the strength of the association between IVPD and protein/kafirin content also markedly reduced with the association becoming non-existent in fermented breads. It is well known that processing flour into different food products alters the structure of the product affecting enzymatic activity towards the proteins. The standard IVPD assay determines only the percentage of the grain protein content digested and does not account for the actual amount of digested protein. When the amount digested is considered, the amount of protein digested per unit of flour/food sample was not very different between genotypes except for the high lysine *Wetet Be-gunchie* and the high tannin *Seredo* in raw flours. For example, 100g of flour from variety *Melekam* (15% protein content) at the reported IVPD of 38% yields 5.7g protein digested. A low protein (10%) cultivar such as *Masugi-Yellow* with a reported IVPD of 61% has a yield of 6.1g protein digested, not very different from the low digestible *Melekam* with the higher total protein. This was the case for all other varieties except the two extremes, *Wetet Be-gunchie* and *Seredo*. The uniqueness with these two genotypes may have to do with their known properties. *Wetet Be-gunchie* has intermediate protein content (13.8%) but low in total kafirin unlike other genotypes of moderately high protein content indicating that the cultivar has relatively higher proportion non-kafirin proteins that are high digestible. Moreover, this genotype also has relatively low γ-kafirin content, a portion of sorghum kafirin known to be resistant to enzymatic degradation. On the other hand, the high tannin genotype *Seredo* is high protein and high kafrin type, the only difference from other genotypes is its high tannin content that perhaps compromised its IVPD both in cooked and uncooked state.

While the strong negative correlation observed between γ-kafirin and IVPD in the raw flour (Fig 4) and a reduction in cooked products may be expected given the reported effect of γ-kafirin [10,70], the complete absence of such association in fermented food product was not clear. The degradation of starch granules and protein during fermentation may increase access for pepsin to more effectively digest protein structures and that the levels of γ-kafirin in raw sample becomes less relevant. The absolute reduction in IVPD of fermented product, however, has to do with the cooking of the protein that may have resulted in altered protein structures through disulfide cross linking or increased hydrophobicity [58,71].

In general, processing cereals into food products reduces the availability of proteins, especially in sorghum and the extent of the reduction depends on both genotype and the type of food product. Even the known high digestible Ethiopian high lysine landrace was not much different from other cultivars when cooked (Table 3) and the reduction has no specific pattern (Table 2) although unfermented wet cooked products have relatively lower IVPD.

Although all genotypes have undergone reduction in IVPD when cooked, the extent to which the reduction occurred was different between genotypes as well as food products resulting in significant genotype by process interaction. The interactions, however, did not seem to follow specific pattern (Table 3). Certain genotypes were better for making fermented bread while others were preferable for making other products. Thus future improvement of sorghum needs to identify grain characteristics that makes them suitable for producing specific food products and direct selection for particular end use traits. However, IVPD is just one trait and final product quality and consumer acceptance also needs to be considered. Moreover, as pointed out elsewhere, IVPD assays do not directly measure the actual amount of protein that becomes available after digestion has taken place (i.e. total digested protein). Another important factor is the difference in gastric emptying time for different foods. Sorghum foods have been reported to have longer stomach emptying time than foods made from other grains [72]. With a longer time in the stomach, proteins from sorghum food may have extended period of exposure to digestive enzymes that the standard pepsin assay may not accurately mimic. If this is confirmed, then protein digestibility may stop to be burning issue in protein nutrition and protein content may become as important if not more. In the current study, the total amount of protein that becomes available after digestion was not different among genotypes especially for cooked products. Hence, in addition to IVPD, attention may be paid to protein content.

Several previous studies have demonstrated considerable differences in percent protein digestibility of raw samples [6,8,9,62,66,69] and industry processed foods [35,37,64,67,73–76]. The current study was also based on raw germplasm samples and food products but deviates greatly from tailored food processing procedures [64, 76]. Many studies that have investigated IVPD of cooked sorghum have used a single 10 or 20 min boiling step of raw flour suspended in water to mimic cooking of porridge [47,62,63,69,77,78]. The current investigation was based on actual foods produced as consumed by local communities in Ethiopia rather than laboratory procedures designed to mimic food production.

### Anti-nutritional factors and mineral content

There was significant effect of genotype and food product effect on anti-nutritional factors (Table 2). Phytic acid content in different food products ranged from 0.62 in fermented bread to 0.77 in raw flour all within the previously reported range of 0.4 and 3.5 g 100g^-1^ [79,80,81]. Food processing seem to have slightly reduced phytic acid levels with fermentation and dry-cooking significantly reduced the compound presumably due to increased phytase activity in fermented and dry-cooked products (Table 5). This agrees with previous studies that showed the effects of cooking and fermentation on phytic acid levels [82]. However, the positive effects of cooking on phytic acid were compromised by increased TIA in all products which along with other chemical changes to the protein body and starch-protein complex may have undermined the overall digestibility in cooked products (Table 3). Adding to this complexity, certain genotypes such as *Wetet Be-gunchie* that have high raw flour digestibility also have higher phytic acid content in all food products (Fig 5). They also have higher phytase activity especially in raw and unfermented wet-cooked products where IVPD from these genotype was higher (Table 3). While TIA was largely not significantly different among high digestible cultivars (Fig 5) certain low digestible varieties such as *Seredo*, or medium digestible ones such as *Teshale* also have similar TIA with that of *Wetet Be-gunchie* and other high digestible cultivars (S1 Table). These all add to the complexity of the sorghum protein digestibility trait.

Similarly, Fe and Zn content across food products was significantly different among genotypes (S2 Table) with cooking appearing to have effect on both minerals (Table 5). However, not all genotypes were affected by cooking with Fe being significantly different between food products for all genotypes except *Dagim*, *Masugi-Yellow*, *IS9302*, *Jigurte* and *Seredo* while Zn content significant only for seven of the fifteen genotypes (S2 Table). Cooking seem to have markedly increased total Fe content with fermentation having the greatest effect (Table 5). While the cause of this increase is not known for certain, it may be the result of the degradation of phytic acid in cooked products and thus release of minerals chelated it. However, the extent of reduction in phytic acid in unfermented food products was very low compared to the increase in total Fe in these products. Nevertheless, similar results have been reported by previous investigators especially of that of fermented products [80,83]. While bioavailability of these minerals was not directly measured in the current project, past studies have used phytate/mineral ratio as potential indicator for bioavailability of both Fe and Zn [42,80]. Using this indicator, bioavailability of Fe and Zn across food products evaluated in the current study ranged from 10–17.9 and 25.1–35.4, respectively (Table 5) with the range for Zn differing slightly from results reported earlier [42]. Due to its high density of negatively charged phosphate groups, phytate binds with minerals and inhibit their availability, especially Fe and Zn bioavailability have been reported to be highly compromised by phytic acid [84–89]. Likewise, a reduction in phytate content improves the availability of these minerals [88,90,91]. Based on the phytate/mineral ratio in the current study, the trend in the availability of Fe and Zn follows the order of fermented bread > dry cooked > wet-cooked > raw flour. Hence, food processing in addition to its effect on IVPD, can influence the availability of essential nutrients and this is of significant practical value to smallholder farmers.

## Conclusions

The study showed that both genotype, food processing method and their intercation have highly significant effect on IVPD of sorghum traditional foods commonly used by rural communities in Ethiopia. The findings shed light on possible avenues towards improving IVPD through manipulating food processing techniques and selection of specific germplasm for making specific food product. Trypsin inhibitor in most of the genotypes was stable and resistant to degradation by food processing. However, processing decreased phytic acid concentration making both protein and minerals more bioavailable.

## Supporting information

S1 Table Trypsin inhibitor activity of genotypes across food processing methods. (PDF)

S2 Table Iron and Zinc content of genotypes across food processing methods. (PDF)
