# Supplementary material for: The effect of genotype and traditional food processing methods on in-vitro protein digestibility and micronutrient profile of sorghum cooked products
Source: PLoS One. 2018 Sep 7;13(9):e0203005. doi: 10.1371/journal.pone.0203005 (PMC6128525; doi:10.1371/journal.pone.0203005)
Supplement: S1 Table — (PDF) [file pone.0203005.s001.pdf]

S1 Table. Trypsin inhibitor activity of genotypes across food processing methods.

Genotype	Average TIA (mg/g)*	Mean separation result for TIA difference among food products‡
Dagim	7.29^{a}	NS
05MI5064	7.13^{ab}	S
76T1#23	7.13^{ab}	NS
Masugi-Yellow	7.03^{a-c}	S
Chiro	6.96^{a-d}	NS
Meko	6.92^{a-e}	NS
Degalite-Yellow	6.92^{a-e}	S
IESV92021-DL	6.89^{a-e}	NS
IS9302	6.83^{a-e}	S
Jigurti	6.73^{b-e}	NS
Melekem	6.67^{b-e}	S
Teshale	6.57^{c-e}	S
Seredo	6.52^{c-e}	NS
Wetet Be-gunchie	6.47^{de}	S
AL-70	6.40^{e}	NS
Mean	6.83	
L.S.D.	0.65	

* Least square means without a common superscript significantly differ at $\alpha = 0.05$; Mean separation results with more than two superscripts are shown as a range using "-" symbol.
‡ Significance of mean separation result; S = significant and NS = non-significant.
