# Supplementary material for: The effect of genotype and traditional food processing methods on in-vitro protein digestibility and micronutrient profile of sorghum cooked products
Source: PLoS One. 2018 Sep 7;13(9):e0203005. doi: 10.1371/journal.pone.0203005 (PMC6128525; doi:10.1371/journal.pone.0203005)
Supplement: S2 Table — (PDF) [file pone.0203005.s002.pdf]

S2 Table. Iron and Zinc content of genotypes across food processing methods*.

Genotype	Average Fe (mg kg^{-1})	Average Zn (mg kg^{-1})	Mean separation result for Fe/Zn difference among food products‡
Dagim	42.6^{b-e}	23.0^{c-e}	NS/NS
05MI5064	41.5^{c-e}	20.4^{d-f}	S/S
76T1#23	50.7^{ab}	25.2^{b-d}	S/S
Masugi-Yellow	47.8^{a-d}	18.8^{ef}	NS/S
Chiro	37.0^{e}	17.0^{f}	S/NS
Meko	52.4^{a}	29.0^{b}	S/NS
Degalite-Yellow	48.4^{a-d}	18.6^{ef}	S/S
IESV92021-DL	43.3^{b-e}	22.4^{c-e}	S/NS
IS9302	40.4^{de}	18.9^{ef}	NS/S
Jigurti	43.0^{b-e}	23.4^{c-e}	NS/NS
Melekem	48.0^{a-d}	29.1^{ab}	S/S
Teshale	49.0^{a-c}	27.1^{bc}	S/S
Seredo	53.4^{a}	28.7^{ab}	NS/S
Wetet Be-gunchie	52.9^{a}	33.6^{a}	S/NS
AL-70	42.9^{b-e}	27.1^{bc}	S/NS
Mean	46.2	24.2	
L.S.D.	9.8	3.5	

* Least square means without a common superscript significantly differ at $\alpha = 0.05$; Mean separation results with more than two superscripts are shown as a range using "-" symbol.
‡ Significance of mean separation result; S = significant and NS = non-significant.
